# Supplementary material for: The effect of music on body sway when standing in a moving virtual environment
Source: PLoS One. 2021 Sep 28;16(9):e0258000. doi: 10.1371/journal.pone.0258000 (PMC8478188; doi:10.1371/journal.pone.0258000)
Supplement: Attachment — Submitted filename: Response to Reviewers.docx [file pone.0258000.s002.docx]

Dear editor and reviewers,

Below is our response to the issue brought forth by Reviewer #2. We appreciate all of the time that both reviewers took to provide thoughtful feedback.

Reviewer #2: There is a small grammatical error on line 121 "Coherence estimates close 1.0" should read: Coherence estimates close to 1.0.

Line 121 has been changed to read “Coherence estimates close to 1.0...”
